# Practical Notes on Diagnosis and Treatment

**Published:** 1906-10-27

**Authors:** 


### Practical Notes on Diagnosis and Treatment.

**Diet in Epilepsy.**

According to Schloss, neither a vegetarian nor a meat diet affects the number of fits in epilepsy.

**Lumbar Puncture in Puerperal Eclampsia.**

Dr. Hebb reports a case of eclampsia in which the convulsions ceased after lumbar puncture. The amount of fluid withdrawn was 38 cubic centimetres. The fluid was free from urea, but reduced Fehling's solution.

**Latent Gastric Cancer.**

The simple continuance of a chronic gastritis or nervous dyspepsia, in spite of logical and scientific treatment, accompanied with progressive loss of body-weight during three or four weeks, justifies the suspicion of latent gastric carcinoma.—*Professor Hemmeter.*

**Corneal Opacities.**

The best method of hastening the clearing of the cornea after an attack of ulceration or of interstitial keratitis is to instil a 5 per cent. solution of dionine every morning and to place a disc of 10 per cent. collargol gelatine in the conjunctival sac every evening.—*Dr. Maitland Ramsay.*

**Local Applications in Erythema.**

As a rule a non-oily preparation is much more satisfactory as a soothing application in cases of erythema than one which contains oil. The following is suitable: Prepared calamine, 15 grs.; zinc oxide, 10 grs.; lime-water, 80 m.; glycerides, 30 m.; water to 1 oz.—*Dr. Jas. Galloway.*

**Anti-pruritic Remedies.**

Of these carbolic acid is perhaps one of the most serviceable; it is best to use it alone well diluted with water or in the form of a dilute glycerine of carbolic acid. The anti-pruritic effects of carbolic acid will be obtained even when the acid is diluted so much as 1 in 100.—*Dr. Jas. Galloway.*

**Treatment of Hæmorrhoids.**

If the patient learns to empty the bowel at bedtime instead of in the morning he gains the chance that the hæmorrhoids when returned within the sphincter will remain there, due to the recumbent posture. This may save much pain. Thorough washing after defæcation should always be practised. As astringents, an injection of 2 drachms of tincture of catechu in 2 ounces of water, or an ointment of equal parts of gall and opium ointment and hazeline cream with 1 per cent. of cocaine are valuable.—*Sir Lauder Brunton.*

**Treatment of Acute Empyema.**

Dr. J. F. Leys urges that in *acute* empyema simple incision without resection of one or more ribs is adequate and successful treatment. In chronic cases, on the other hand, sufficient drainage may be impossible without removal of bone.

**Electricity in Functional Enuresis.**

Faradisation of the bladder for five or ten minutes, with gradually increasing current, has been found useful in this condition. Either the two electrodes are placed over the bladder or one over the bladder and the other on the sacrum or perineum.

**Rest and Poultices in Chronic Gastritis.**

Dyspeptics are not sent to bed often enough. Rest in bed facilitates the treatment in every way. It enables the patient to get on comfortably with far less food, it keeps him at a uniform temperature, and it facilitates the use of local applications. Of these we do not make, in such cases, sufficient use of moist heat. When the patient is kept in bed continuous poulticing may be adopted for a week, whilst in milder cases a hot fomentation may be applied at night and an abdominal flannel belt worn during the day.—*Dr. Robert Hutchison.*

**Treatment of Iritis, etc.**

Mr. Chas. Wray advocates the administration of acetozone in iritis, keratitis, and other intraocular inflammations. He orders a capsule containing three grains of the drug four times daily. The dose is immediately preceded by a tumblerful of water, and is followed by walking exercise continued for ten minutes. After this a second tumblerful of water is taken and the exercise repeated. The results are described as "admirable" in all cases in which the patient was seen within a week of the commencement of the disease.

**Minced-meat Diet.**

I have repeatedly assured myself of the value of this in the treatment of serious cases of dyspepsia where milk has not been tolerated. The meat must be lean, and may be mutton or beef. It should be minced before cooking, but need not be eaten half raw. I recommend it to be cooked in the stewpan with sufficient water to make it easy to eat. No condiments should be allowed with it, and if there is reason to suspect excessive acidity or hypersecretion of gastric juice even salt should be forbidden.—*Dr. Robert Saundby.*

